# Sphingomyelin-Rich Lipid Extract Collar for Canine Atopic Dermatitis

**DOI:** 10.3390/vetsci10060389

**Published:** 2023-06-07

**Authors:** Sergi Segarra, David Sanmiguel, Eliseo Zuriaga, Sophie Leclerc, Jesús Cabañas, Estelle Seigneuric, Aurélie Miquel, Ana Vázquez, Lluís Ferrer

**Affiliations:** 1 R&D Bioiberica S.A.U., Avinguda dels Països Catalans, 34, 08950 Esplugues de Llobregat, Spain; jcabanas@bioiberica.com; 2 Clínica Veterinaria Wecan De Carreres, Avinguda Mutxamel, 1, Local 2, 03550 Sant Joan d’Alacant, Spain; decarreres@clinicaswecan.com; 3 Hospital Veterinario Global, Carrer Laminacio, 18, 46520 Port de Sagunt, Spain; dermanimalvalencia@gmail.com; 4 AB7 Santé, BP 9 Les Monges, 31450 Deyme, Francee.seigneuric@ab7innovation.com (E.S.); a.miquel@ab7innovation.com (A.M.); 5 Servei d’Estadística Aplicada, Facultat de Ciències, Universitat Autònoma de Barcelona, C5b/111, Edifici C, 08193 Cerdanyola del Vallès, Spain; ana.vazquez@uab.cat; 6 Departament de Medicina i Cirurgia Animals, S/N, Campus Universitat Autònoma de Barcelona, 08193 Cerdanyola del Vallès, Spain; lluis.ferrer@uab.cat

**Keywords:** atopic dermatitis, sphingolipids, sphingomyelin, collar, pruritus, atopic disease, new technologies, feline dermatology

## Abstract

**Simple Summary:**

Canine atopic dermatitis (CAD) is a chronic skin disease which can be very frustrating for veterinarians and pet guardians. Adherence to treatment is key when managing CAD cases. For this reason, we developed and tested a new product for CAD (Atopivet^®^ collar, Bioiberica SAU, Esplugues de Llobregat, Spain) which is easy to administer and could help reduce monthly treatment costs. This novel product is a plastic collar containing a lipid extract (Biosfeen^®^, Bioiberica, S.A.U., Esplugues de Llobregat, Spain) which has been studied previously and has been shown to have beneficial effects on skin health. The studies reported in this article show how this lipid extract is adequately released when incorporated into a collar presentation. They also demonstrate the collar’s good efficacy and safety after evaluating its effects in a multicentric, prospective, uncontrolled pilot study in client-owned dogs with naturally occurring CAD. It is concluded that this collar can become a useful therapy to be considered within a multimodal approach to CAD, providing veterinarians with more tools in order to define the best tailor-made approach to each particular case, as well as providing pet guardians with a solution that could eventually make their lives easier.

**Abstract:**

The management of canine atopic dermatitis (CAD) is complex, and it needs to be multimodal, combining topical and systemic therapies. Given that the currently available options are not always totally effective and might have some associated adverse effects, novel alternatives are needed. For this reason, a new collar for CAD was developed with 2.5% of a sphingomyelin-rich lipid extract (LE) with proven benefits for skin health. The release of the active ingredient when incorporated into the collar was tested in vitro, showing an adequate kinetic profile. Then, the efficacy and safety of the collar were assessed in 12 client-owned dogs with CAD in a pilot study. After eight weeks, the dogs experienced significant clinical improvements on the Canine Atopic Dermatitis Extent and Severity Index (CADESI)-4, Pruritus Index for Canine Atopic Dermatitis (PCAD) and Pruritus Visual Analogue Scale (PVAS) scores, without any adverse effects. Additionally, further in vitro studies were performed, indicating that this LE collar should be compatible with antiparasitic collars (with deltamethrin or imidacloprid/flumethrin) if worn simultaneously. Given the observed benefits of this LE collar, combining it with other CAD therapies could potentially allow for drug sparing, reduction in adverse effects, enhanced owner compliance, and reduced treatment costs.

## 1. Introduction

Canine atopic dermatitis (CAD) is a genetically predisposed, progressive, chronically relapsing, inflammatory, and pruritic skin disease with characteristic clinical features associated with immunoglobulin (Ig)E antibodies most commonly directed against environmental allergens. The main clinical sign of CAD is pruritus, which is also currently the main complaint in canine dermatology, and which affects the quality of life of dogs and their owners [1,2,3,4].

The management of CAD follows a multimodal approach and the treatment strategy required for CAD combines topical and systemic therapies, which need to be tailored to each individual dog and owner situation [1,2,5]. Several options are available when it comes to topical therapy, including shampoos, foaming mousses, sprays, wipes, spot-on treatments, creams, and ointments [1,6] but, unfortunately, the currently existing therapies are not always totally effective, and might have some associated adverse effects [7].

Epidermal barrier dysfunction plays a key role in the pathogenesis of CAD, as it allows microbial adherence, penetration of allergenic proteins, and may trigger inflammatory and allergic responses. That is why one of the targets of topical therapy in CAD patients is to restore epidermal barrier integrity and function [1,3,8,9,10,11,12]. Currently, the management of the atopic canine patient includes restoration of the skin barrier function by, for instance, applying sphingolipids and fatty acid emulsions [2,13]. Nonetheless, at present, CAD can often become a frustrating disease for both veterinarians and pet guardians [14,15]. For this reason, novel treatments, and also novel presentations, are required in order to provide vets with different treatment alternatives.

Sphingolipids are key elements in the plasma membrane of the eukaryotic cells, and, in the epidermis, they contribute to the skin barrier function. Ceramides are the main epidermal sphingolipids, and lower levels in the skin may lead to skin barrier dysfunction in dogs and in people [10,16,17,18]. Several in vitro studies support the beneficial effects on skin health of a sphingomyelin-rich lipid extract (LE; Biosfeen^®^, Bioiberica, S.A.U., Esplugues de Llobregat, Spain). In an in vitro model of skin equivalents, the application of this LE led to increased levels of ceramides and the number of lamellar bodies [19], and, in a reconstructed human epidermis (RHE) in vitro model, this LE achieved an enhancement of the expression of filaggrin and also of the expression of antimicrobial peptide (AMP) human β-defensin 2 (hBD-2) [20]. Moreover, in vivo, this LE has been proven to provide clinical benefits in dogs with atopic dermatitis when applied topically in combination with a glycosaminoglycan matrix [21], and it has been included as a topical therapy option to restore epidermal barrier integrity and function [1,2].

Enhancing pet guardians’ adherence to treatment is of paramount importance when managing CAD cases, and this may be achieved by facilitating product administration, reducing treatment costs, and covering different preferences. With the aim of contributing to better management of CAD patients by pet guardians and veterinarians, a novel collar product was developed and tested in vitro and in vivo with the aim of evaluating its potential usefulness for the management of CAD. Our hypothesis was that this collar presentation could allow a more sustained and gradual release of the active ingredient.

Our objective was to study the potential usefulness of the abovementioned sphingomyelin-rich LE collar for CAD by evaluating its in vitro release kinetic pattern and its in vivo efficacy and safety in atopic dogs.

## 2. Materials and Methods

### 2.1. Lipid Extract Collar

**Figure 1:**
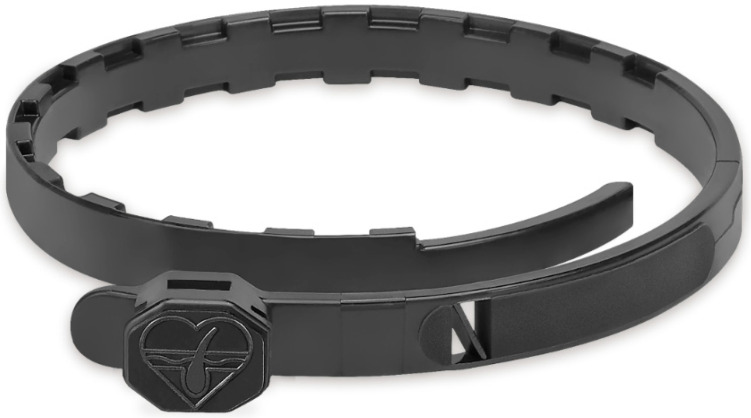
Photograph of one of the sphingomyelin-rich lipid extract collars developed for the management of atopic dermatitis in dogs.

A collar product (Atopivet^®^ collar, Bioiberica SAU, Esplugues de Llobregat, Spain) was developed using a polymer (TPU, thermoplastic polyurethane) matrix collar containing 2.5% LE Biosfeen^®^. The collar also incorporates 1.5% lavender oil to provide an appropriate smell, a plasticizer (2-ethylhexyl diphenyl phosphate) which provides flexibility to the collar and enhances the release of the active ingredient, and a black dye in order to protect the active ingredient from UV light (Figure 1). This collar was developed to be used, as its main indication, in dogs with atopic dermatitis.

The technology used in this new product development as well as its chosen design were selected in order to ensure an adequate release of the active ingredient from the TPU matrix and also a steady and sufficient diffusion of the LE on the skin surface. More specifically, the plasticizer opens the polymer network, which allows the release of the LE. Afterwards, when the collar has been placed around the dog’s neck, the LE is distributed and spread across the *stratum corneum* through the lipids of the skin, taking advantage of the lipophilic nature of the LE. The epidermal lipids are therefore expected to act as a vehicle to allow the active ingredient to advance over the surface of the skin.

### 2.2. Evaluation of In Vitro Release Kinetic Pattern

In order to evaluate the in vitro release kinetic pattern of the LE when incorporated into the polymer matrix collar, three collars were fragmented into several pieces. The fragments were immersed in a fatty medium containing a mixture of triglycerides and shaken with a magnetic stirrer. The LE content was obtained using methyl palmitate measurements from the samples, which were quantified using gas chromatography with flame-ionization detection (GC-FID), first at baseline, and then after 8, 15, and 22 days. These results were used to determine the release percentage of the LE over time.

### 2.3. Efficacy and Safety in Dogs with Atopic Dermatitis

After the in vitro test, the clinical efficacy and safety of this collar were evaluated in a multicentric, prospective, and uncontrolled pilot study in client-owned dogs with naturally occurring CAD. Dogs of any age, sex, and breed were assessed for eligibility if they presented with a history and clinical signs compatible with non-seasonal CAD, after other causes of pruritic dermatitis had been ruled out. CAD diagnosis was made following the criteria of Favrot et al. [22]. Dogs whose sole clinical sign was otitis, or gestating or lactating females were not included. Dogs could not be included either if they were being given a diet specially indicated for the management of skin health, if they had received fatty acids by any route eight weeks prior to the study onset, or a product from the Atopivet^®^ range (Bioiberica, S.A.U., Esplugues de Llobregat, Spain) by any route two weeks prior to the start of the study, or if they had been administered systemic glucocorticoids, oclacitinib, lokivetmab, or cyclosporine in the last four weeks before inclusion in this study. Shampoo baths were allowed to manage bacterial or *Malassezia* infections, as long as they involved using 3% chlorhexidine or 2% miconazole with 2% chlorhexidine, and without phytosphingosine. Pyoderma, *Malassezia* dermatitis, and ectoparasites were ruled out before inclusion, based on a physical examination, cytology, and negative skin scrapings, in addition to the lack of response to antiparasitic treatment (insecticide and acaricide) carried out at least during the two months preceding inclusion. All eligible candidates also had to undergo an elimination diet for a minimum of eight weeks before inclusion to rule out adverse food reactions. Dogs were excluded from the study if they showed evident signs of intolerance or adverse effects to the treatment applied, or a severe worsening of clinical signs.

One polymer matrix LE collar was applied around the neck of each of the dogs included in this study and left on for eight weeks. The Canine Atopic Dermatitis Extent and Severity Index (CADESI)-4 [23] and Pruritus Index for Canine Atopic Dermatitis (PICAD) [24] were assessed by the veterinarian at baseline and after four and eight weeks. The CADESI-4 is a tool that can be used to classify the cases as mild (10–34 points), moderate (35–59 points), or severe (≥60 points) based on the severity and extension of the lesions. The PICAD quantifies the degree of pruritus (from 0 to 64) according to the frequency and intensity in different anatomical areas. On the other hand, the Pruritus Visual Analogue Scale (PVAS; 0 to 10) was assessed weekly by the pet guardians.

The variables of interest were described using mean and standard deviation (mean ± SD). The comparison of the variables between time points has been carried out using a mixed model with animal as a random factor. The *p* values of the comparisons between time points were corrected by Tukey’s correction method. The significance level was set at 0.05 in all tests. The results were analyzed using SAS v9.4 software (SAS Institute Inc., Cary, NC, USA).

### 2.4. Analyses of Used Collars

With the aim of characterizing the release of the LE when applied to the dogs, and in order to be able to correlate the findings of the in vitro release kinetic study with those of the in vivo study, collars belonging to six cases from the study were analyzed before and after use. More specifically, laboratory analyses were performed on samples of collars after being worn by the dogs for eight weeks and compared to the results of the same analyses before applying the collars on the dogs. Duplicates of 300 mg of collar samples were placed for 4 h at 48 °C in 5 mL of a 2:1 methanol/chloroform mix. Thereafter, 0.05 mL of each sample were saponified in KOH in methanol and injected for lipid analysis via ultra-high performance liquid chromatography coupled to time-of-flight mass spectrometry (UPLC-TOF) to measure total sphingolipids and lipid profiles.

### 2.5. Compatibility Studies

Given that the use of antiparasitic collars is widespread in client-owned dogs, it was deemed necessary to ensure the compatibility of the LE contained in this novel collar product with the active ingredients found in commonly used antiparasitic collars. For this reason, the compatibility of the LE with imidacloprid/flumethrin (as found in Seresto^®^, Elanco Animal Health, Greenfield, IN, USA) [25,26,27,28] and with deltamethrin (as found in Scalibor^®^ protector band, MSD Animal Health, Rahway, NJ, USA) [29,30,31] were assessed in order to account for any potential interactions. A mix containing 10% imidacloprid, 4.5% flumethrin, 2.5% LE Biosfeen^®^, 2.0% lavender oil, and 81% 2-ethylhexyl diphenyl phosphate was prepared and, after stirring, stored in flasks for 2 weeks at 54 °C. The same procedure was also performed with another mixture containing 4% deltamethrin, 2.5% LE Biosfeen^®^, 2.0% lavender oil, and 91.5% 2-ethylhexyl diphenyl phosphate. The LE content quantified using GC-FID for the in vitro release kinetic studies, and deltamethrin, imidacloprid, and flumethrin were quantified using high performance liquid chromatography (HPLC). Recovery percentages for all substances were calculated by working out the difference between the final (test results) and initial (theoretical values) content in the samples.

## 3. Results

### 3.1. In Vitro Release

**Figure 2:**
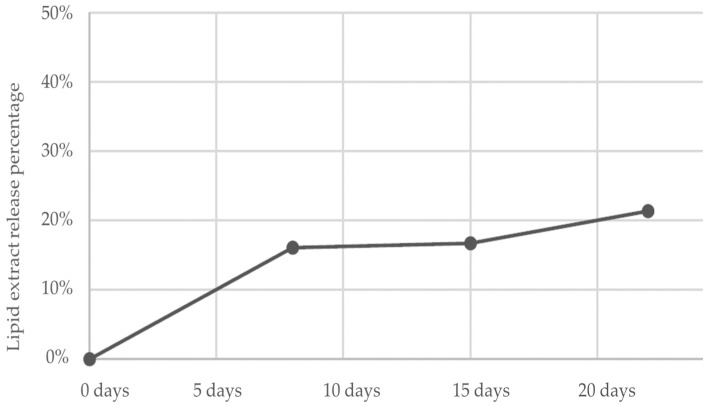
Percentage of release of the lipid extract in collar samples analyzed with GC-FID at baseline, and after 8, 15 and 22 days.

The mean LE levels obtained from the methyl palmitate content remaining in the collar fragments after 0, 8, 15, and 22 days was, respectively, 2.56%, 2.15%, 2.13%, and 2.01%. This represents a release of 0%, 16%, 17%, and 21%, respectively, from the original content (Figure 2).

### 3.2. In Vivo Efficacy and Safety

A total of 12 dogs were included in the study, none of them were lost during the follow-up, and they all completed the study and wore the LE collar for eight weeks. This included five males and seven females (73.17 ± 41.74 months of age) of different canine breeds: crossed breed (*n* = 5), Labrador retriever (*n* = 1), German shepherd (*n* = 1), hound (*n* = 1), shiba inu (*n* = 1), Shi Tzu (*n* = 1), wire-haired dachshund (*n* = 1), and West Highland white terrier (*n* = 1). No adverse effects were observed in any of the dogs during the course of the study.

**Figure 3:**
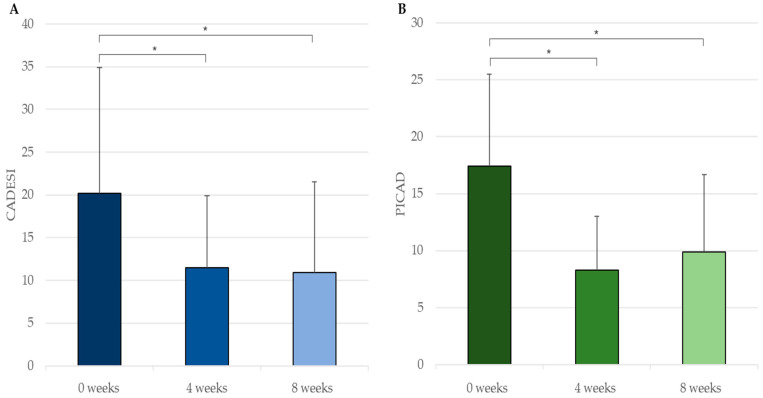
(**A**) CADESI-4 (mean+SD) and (**B**) PICAD (mean+SD) evaluations by the veterinarians at 0, 4 and 8 weeks. * *p* < 0.05.

**Figure 4:**
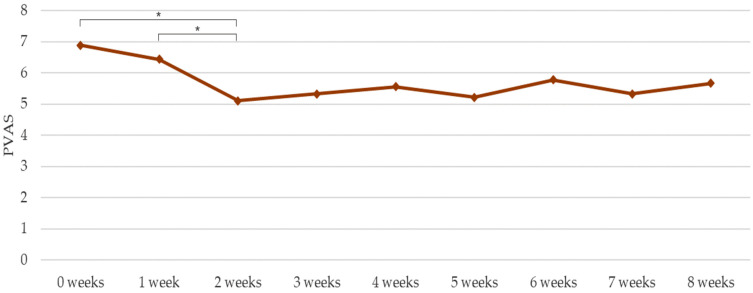
PVAS (mean) weekly evaluations by the pet guardians. * *p* < 0.05.

Through an ANOVA model with repeated measurements, statistically significant reductions (*p* < 0.05) were observed in CADESI-4 from zero (20.17 ± 14.73) to four weeks (11.50 ± 8.41; 42.98% decrease) and from zero to eight weeks (10.89 ± 10.67; 46.01% decrease); in PICAD between zero (17.42 ± 8.08) and four weeks (8.30 ± 4.71; 52.35% decrease) and between zero and eight weeks (9.89 ± 6.79; 43.22% decrease) (Figure 3); and in PVAS from zero (6.89 ± 1.17) to two weeks (5.11 ± 2.31; 25.83% decrease) and from one (6.44 ± 1.51) to two weeks (20.65% decrease) (Figure 4).

**Figure 5:**
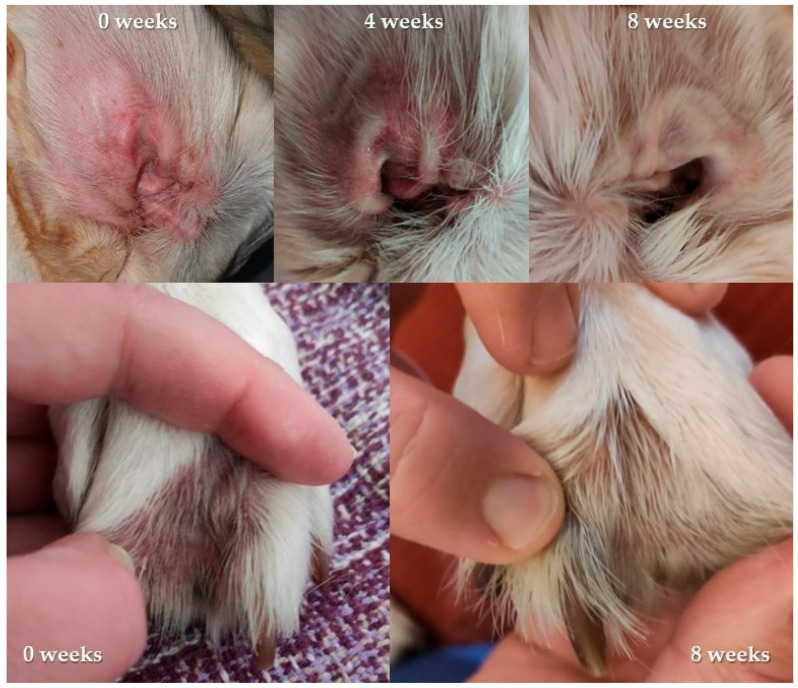
Representative images of the inner part of the pinnae (top images) and interdigital space (bottom images) in a Labrador retriever from the study after 0, 4, and 8 weeks.

Clinical improvements were observed over time in several areas, including distal parts of the body (Figure 5).

### 3.3. Analysis of Used Collars

The variations in the lipid profile analyzed by UPLC-TOF in the LE collars before and after 8-week application in CAD patients are shown in Table 1.

### 3.4. Compatibility with Imidacloprid, Flumethrin and Deltamethrin

When theoretical values and actual results from the assays were compared, recovery percentages were 101.0% for LE, 101.0% for imidacloprid, and 100.0% for flumethrin in the first mixture; and 93.0% for LE and 95.5% for deltamethrin in the second mixture.

## 4. Discussion

The topical application of lipid-based formulations aimed at improving skin barrier dysfunction has been previously investigated in several studies in dogs [32,33,34,35,36,37,38,39,40,41,42,43]. These interventions have shown some positive effects on skin health, and they are normally delivered in the form of a spot-on or a shampoo presentation. Incorporating a LE into a polymer matrix and applying this in a collar presentation represents a new approach to acting on skin lipids [12], and as a more user-friendly way of managing CAD patients. The results described in the in vivo test in CAD dogs support the efficacy and safety of such a LE collar. Significant benefits were seen on the CADESI score as well as on the evaluation of pruritus, which is the main clinical sign of CAD [44], and for which both vets and pet guardians reported significant improvements with the tested product.

The pilot study reported in this article shows significant improvements in several clinical parameters. It is also worthwhile mentioning that these benefits were achieved based on observations made both by the veterinarian and the pet guardian. Indeed, the CADESI and PICAD scores reflected clinical improvements in comprehensive evaluations made by the clinicians, while the PVAS indicated that pet guardians also perceived such improvements from their subjective standpoint. In this regard, it should also be pointed out that a marked decrease in all three scoring systems was noted, especially during the first two to four weeks. After that, although no significant clinical worsening is observed, the graphs show a plateau, and the score values remain relatively stable up until the end of the eight-week follow-up. Perhaps in a clinical setting, and based on these results, it would be advisable to recheck the patient one month after the first application of the LE collar in order to evaluate whether it needs to be replaced by a new one or whether other therapies are warranted in addition to such a collar.

When managing CAD patients, it is important to educate pet guardians so that they understand that CAD is a lifelong and progressive disease, and that it requires long-term management. With that, realistic expectations can be set, and it might be easier to increase compliance and reduce frustration over time [1,2,15]. Keeping the pet guardian fully engaged might be challenging, but it may be more critical even than treating the CAD patient, which is why novel and more convenient product presentations may be well perceived by vets and pet guardians. The approach to CAD has evolved over time and nowadays, rather than using a reactive approach, a proactive approach is encouraged by veterinary clinicians facing CAD cases. This means acting with the aim of preventing flares instead of waiting for them to occur and then treating the patient accordingly [2,13]. The concept of using the LE collar described herein, together with its benefits described in this article, would fit within such a proactive and multimodal strategy to manage CAD. It might become especially useful as a complementary tool for managing the chronic phase of the disease in cases where frustration stems from a management strategy that turned out to be inadequate.

Taking into consideration the type of subjects enrolled in the study and the severity and degree of pruritus at baseline, and with the information available at this point, this product should be indicated especially for the management of mild to moderate CAD cases. In more severe clinical cases, or when pruritus frequency or intensity are also high, antipruritic therapies should be used. In such situations, this LE could serve as a drug-sparing agent, allowing a reduction in the use or dosage of other therapies and potentially also leading to fewer associated side effects.

One of the key points during the process of developing this novel product was ensuring an adequate release and distribution of the active ingredient. The results of the in vitro study reported herein support the release of the LE in an in vitro setting which emulates what occurs in vivo. The observed release profile is similar to that of other active substances commonly incorporated in antiparasitic polymer matrix collars (AB7 Santé internal data) which then diffuse correctly in vivo once applied to dogs. In fact, when collars were analyzed in vivo before and after having been used for eight weeks, the percentage of release of the active ingredient was similar to that of the one obtained in vitro, and the ratio between different type of lipids was also maintained when pre- and post-measurements were compared. Moreover, the clinical improvements seen in distal body parts further indicate that the distribution of the LE takes place thanks to the above-mentioned technology. Regarding the sphingolipid profile determined by UPLC-TOF, it should be mentioned that, although total sphingolipids and most lipid components decreased overtime as expected, the levels of dihydroceramides were surprisingly higher after eight weeks, compared to baseline. However, even if a 25% increase was observed in this lipid component, such a rise in dihydroceramides levels represents only 0.0025%, from 0.01000 to 0.01250% in the total composition of the collar. This value is relatively smaller compared to those of other components and the magnitude was not considered relevant.

The use of this collar might facilitate the management of CAD, but vets might worry about a potential interaction with other collars. Most dogs normally wear antiparasitic collars and the use of the sphingomyelin-rich LE collar described in this article could have an impact on its efficacy, and vice versa. Compatibility studies with imidacloprid/flumethrin and deltamethrin were performed to rule out any potential interferences between these different types of collars and that of their active ingredients. Having done this in a setting where the active ingredients are mixed together under intensive conditions, and given the satisfactory results, no problems are expected with regards to animals wearing an antiparasitic collar and the LE collar together.

Despite the data presented herein, at this point there are some limitations and further investigations are still required. The in vivo study provides interesting efficacy and safety data, but the lack of a control group makes it less robust. For this reason, evaluating the effects of this new product development in the setting of a properly designed randomized controlled clinical trial would be desirable. This would be helpful in order to confirm a correlation between the observed changes in disease outcomes and a clear treatment effect of this product. On the other hand, although we studied the release and distribution of the active ingredient across the skin, and even though our data show positive results, further investigation would be required in order to better characterize to what extent the LE is released from the collar, as well as how precisely it binds to the *stratum corneum* lipids, how it is transported to the rest of the body regions, and how it impacts objectively on lesions found in distal parts of the body.

Nevertheless, and although further investigations and a longer-term experience from its use in clinical practice are needed, this collar could become a useful tool within the multimodal management approach to CAD. Furthermore, and based on the findings reported in this article, a potential path to be explored in the future would be the usefulness of this LE collar for managing cats suffering from feline atopic skin syndrome (FASS). The understanding of this disease is still not as advanced as that of CAD, but similarities exist with regards to its pathogenesis, clinical signs, and treatment options [45,46,47,48]. So far, a case report describes the clinical benefits and safety of this LE when used in a cat with FASS [49], leading to significant improvements in the SCORing Feline Allergic Dermatitis (SCORFAD) scale and the PVAS. However, the performance of clinical trials would be necessary to confirm these observations.

## 5. Conclusions

A new collar for CAD has been developed incorporating a LE backed up by prior scientific evidence supporting its benefits for skin health. The technology used for manufacturing this innovative collar allows for a good release of the LE. When tested in vivo in CAD patients, this collar provides significant clinical benefits on the CADESI, PICAD and PVAS scores. Moreover, the in vitro tests indicate that it should be compatible with antiparasitic collars if worn simultaneously. Given the observed benefits of this LE collar, combining it with other CAD therapies could potentially allow for drug sparing, reduction in adverse effects, enhanced pet guardian compliance, and reduced treatment costs.

## Figures and Tables

**Table 1:** Sphingolipids profile determined by UPLC-TOF in collar samples and variations between 0 and 8 weeks.

Component	Mean % 0 Weeks	Mean % 8 Weeks	Variation
Ceramides	0.14833	0.11250	−24.16%
Dihydroceramides	0.01000	0.01250	+25.00%
Sphingomyelins	2.64833	2.20000	−16.93%
Dihydrosphingomyelins	0.14833	0.13625	−8.15%
Glycosylceramides	0.06167	0.04875	−20.95%
Lactosylceramides	0.13667	0.10750	−21.34%
Total sphingolipids	3.15330	2.62130	−16.87%

## Data Availability

The datasets used and/or analyzed during the studies reported herein are available from the corresponding author on reasonable request.
